# Is it possible to gain energy at work? A questionnaire study in primary health care

**DOI:** 10.1017/S1463423620000614

**Published:** 2020-12-17

**Authors:** Lina Ejlertsson, Bodil Heijbel, Annika Brorsson, H. Ingemar Andersson

**Affiliations:** 1 Faculty of Medicine, Department of Clinical Sciences in Malmö, Lund University, Malmö, Sweden; 2 Faculty of Health Science, Kristianstad University, Kristianstad, Sweden

**Keywords:** energy, health promotion, healthy work conditions, occupational health, primary health care, salutogenic

## Abstract

**Objectives:**

The area of regenerative work is still close to unexplored. The aim was to explore the possibility for employees to gain energy at work.

**Methods:**

Questionnaire to all employees (*n* = 599) from different professions in public and private primary health care centers in one health care district in Sweden. The questionnaire, which had a salutogenic perspective, included information on self-rated health, psychosocial work environment and experiences, recovery, social climate, and energy. Having an energy-building experience was defined by a positive response to two combined questions regarding energy at work. Analyses were performed with bivariate correlation and multiple logistic regression.

**Results:**

The response rate was 84%. Health and energy correlated positively (*r* = 0.54). In total, 44.5% of the employees reported having an energy-building experience. Predictors for having an energy-building experience were recovery [positive odds ratio (POR) = 2.78], autonomy (POR = 2.26), positive workplace characteristics (POR = 2.09), and internal work experiences (POR = 1.88).

**Conclusions:**

The results support the hypothesis that it is possible to gain energy at work, an area that is still close to unexplored. There is a high correlation between energy and health. Employees’ energy-building experiences relate to well-being at work and correlates to recovery, autonomy, positive workplace characteristics, and positive internal work experiences. This knowledge can help in improving future work environment development.

## Introduction

Work can influence individuals in both positive and negative ways. Knowledge of health related to the work situation has gradually developed during the last 150 years (Gochfeld, 2005), from chemical exposure, accidents, living conditions and poverty, to preventive measures (Quick, 1999), and workplace health promotion. The latter emanated from the Ottawa Charter on health-promoting arenas (World Health Organization, 1986) and is considered by the World Health Organization as a prioritized domain into the 21st century, since work is an essential setting for the promotion of health (World Health Organization, 2019).

The dominant paradigm in work environment research is still mainly pathogenic. This means that the focus is on risk factors causing ill-health (Eriksson and Lindström, 2008). Job demands, such as having a stressful job with a heavy workload, are directly linked to, and have a negative impact on, employees’ perceptions of the extent to which their workplace is healthy (Lowe *et al.*, 2003). Primary health care is an example of such a work context (Teles *et al.*, 2014). But in recent years, the focus has been transferred from shortcomings, stressors and illnesses, towards research with a more salutogenic, that is positive, approach (Kelloway *et al.*, 2008; Jenny *et al.*, 2016). The concept of salutogenesis sets out from the healthy and from the resources in human beings and environments, which can lead to improved health (Antonovsky, 1987). Having a strong sense of coherence (scoring high on comprehensibility, meaningfulness, and manageability) (Antonovsky, 1993) decreases the possible negative effects of work stressors (Eberz *et al.*, 2011) and may reduce the risk of sick leave (Kuoppala *et al.*, 2011). The way that we view the world affects our capability to cope with both tension and stress (Eriksson and Lindström, 2007), and positive emotions help us to manage when we are confronted with negative situations (Uncu *et al.*, 2007).

A further development of health promotion points out the possibility of work being an internal source of energy for the individual, so-called regenerative work (Kira, 2003; Ericsson, 2010). Regenerative work is about work being a positive contributor to the individual’s energy, development, and job satisfaction. Work that is characterized by being regenerative supports the development and regeneration of employees’ cognitive and emotional resources (Kira and Forslin, 2008). Thus, regenerative work means that work also can recreate spent human resources (Kira *et al.*, 2008; Palm, 2008).

Energy is a complex concept with many nuances. In the literature, different types of energy are described, including mental energy (Balk *et al.*, 2019), physical energy (Ampel *et al.*, 2018), emotional energy, relational energy, and organizational energy (Baker, 2019). Employees’ experiences of energy at work may be summarized as the variation of various individual resources throughout the workday.

There is a lack of information about the experience of energy at work, as well as the potential of work to recreate human resources, and in the next step if and how energy affects health. The aim of this study was to explore the possibility to gain energy at work. What are the predictors for employees’ *energy-building experiences*? Is there any relationship between energy and health?

## Material and methods

### Setting and participants

A cross-sectional questionnaire study was conducted in public and private primary health care centers (PHCCs), comprising both urban and rural areas. All 26 PHCCs in one health care district in southern Sweden participated. Primary health care is the base of Swedish health care outside the hospitals, but accounts for only 17% of the total health care expenditure. In the PHCCs various professionals collaborate, such as physicians, nurses, psychologists, physiotherapists, and medical secretaries. The mental and emotional demands are high and similar for all professional groups. Since primary health care in Sweden only includes out-patient care, the physically demanding work is limited. All employees (*n* = 599) of different professions were invited to participate. Staff on long-term sick leave or maternity leave was excluded, as well as all the managers and owners of the PHCCs.

### Questionnaire and procedure

The questionnaire had a salutogenic perspective and was based on two validated instruments: SHIS (Salutogenic Health Indicator Scale), measuring indicators of self-rated health (Bringsén *et al.*, 2009) and WEMS (Work Experience Measurement Scale) (Nilsson *et al.*, 2010). WEMS describes employees’ experiences regarding the psychosocial work environment, for example influence over work situation, good leadership, support from co-workers, and feelings of happiness when going to work. Further questions on energy, reflection, and recovery at work supplemented these measures, together with questions on general self-esteem and optimism and recovery outside of work. They were mainly developed through analyses of five focus group interviews and four individual interviews in some of the participating PHCCs (Ejlertsson *et al.*, 2018). The questionnaire consisted of 25 main question areas with a total of 121 items. Concepts like energy and recovery were not further defined in the questionnaire, due to the complexity of their various facets. Instead, the respondents were allowed to interpret them based on their own frame of reference.

For most of the questions, a symmetric Likert-type scale was used, where the respondents specified their level of agreement or disagreement. The statements were positively phrased, with six response alternatives ranging from *totally agree* to *totally disagree*. In two of the question groups, a semantic differential with six steps was used.

Questions on age, sex, profession, and employment rate were included in the questionnaire. The different professions were physician, nurse (registered nurse, assistant nurse), paramedical staff (psychologist, counselor, occupational therapist, physiotherapist, dietician), and administrative staff (such as medical secretary and receptionist).

For questions besides the previously validated SHIS and WEMS, content validity was addressed by an expert panel, who asserted that the items in the questionnaire reflected the knowledge base. To enhance the face validity of the study, a pilot study was conducted. Different professions in primary health care completed the questionnaire, while commenting on the understanding of, and their responses to, the questions. This ‘think-aloud interviewing’ (Charters, 2003) resulted in some minor changes of the questionnaire, which was distributed in the autumn of 2013.

The first author attended work group meetings in 16 of the centers, and the employees completed the questionnaire on the spot. Absent employees were given the questionnaire and a prepaid reply envelope afterwards by the manager. In the remaining 10 centers, the manager distributed the questionnaires to the employees. For confidentiality reasons, a comprehensive reminder to all employees was issued after a couple of weeks instead of personal reminders.

### Analyses

Besides the already existing SHIS and five sub-indices of WEMS, 10 more indices were constructed, 8 of which were used in the regression model (Table 1). The subject areas of the indices were decided on theoretical and empirical grounds. The reliability, that is the internal consistency, of the indices was calculated with the Cronbach’s alpha (CA) coefficient (Cronbach, 1951). To be accepted as an index, the CA coefficient had to be higher than 0.70 (Bland and Altman, 1997).

**Table 1.** Presentation of the indices used in the study

Index	No. of questions	Scale type	Example of questions	Cronbach’s alpha	Score min–max (mean; median)
Energy at work	3	Likert-type scale	I feel that … my job gives me new energy, … the energy I get in my work exceeds what I expend, … we give each other energy between colleagues	0.80	3–18 (11.8; 12)
Recovery	4	Likert-type scale	I feel I get time for recovery … during working hours, … outside work	0.83	4–24 (15.4; 16)
Attributes that characterize the workplace	8	Semantic differential	Characteristics which reflect my workplace. Positive/negative, safe/unsafe, open/closed	0.96	8–48 (35.8; 37)
Work situation	6	Likert-type scale	I feel that … we share the same basic values at my workplace, we respect each other at my workplace, … I am secure in my professional role	0.81	10–36 (24.4; 24)
Feedback	3	Likert-type scale	I get feedback on the work I do, the manager gives me feedback about the work I do	0.80	3–18 (12.6; 13)
Reflection	3	Likert-type scale	I take time to reflect on work-related events, Self-reflection helps me see my job as meaningful	0.73	5–18 (13.9; 14)
Relationship with co-workers	3	Likert-type scale	I try to be an inspiration to my colleagues, I feel that my colleagues trust me	0.82	5–18 (14.2; 14)
Expectations	4	Likert-type scale	I can achieve what is expected of me at work, My colleagues would say that I meet their expectations	0.85	6–24 (19.6; 20)
Self-esteem and optimism	11	Likert-type scale	I try to see the positive aspect in events in my life, Other people respect me, I feel that am a valuable person	0.93	17–66 (55.4; 56)
SHIS	12	Semantic differential	In the last 4 weeks, I have… felt alert/felt tired, felt happy/felt sad	0.94	12–72 (51.8; 52)
Supportive working conditions (WEMS)	7	Likert-type scale	We encourage and support each other at work, I get feedback on the work I do	0.90	9–42 (31.0; 32)
Internal work experience (WEMS)	6	Likert-type scale	I feel that my work is meaningful, I am happy when I go to work	0.86	7–36 (29.9; 31)
Leadership (WEMS)	6	Likert-type scale	My boss is available when I need him/her, my boss helps us divide our work in a fair way	0.92	6–36 (26.5; 28)
Time experience (WEMS)	3	Likert-type scale	I have enough time during my normal working hours to do my job without time pressure (stress)	0.87	3–18 (10.4; 11)
Autonomy (WEMS)	4	Likert-type scale	I decide when to do the various work tasks, I decide how to do my work	0.85	4–24 (14.8; 15)

The relations between all quantitative variables were evaluated by Pearson bivariate correlations. From the combination of positive responses (6–4) to two questions (*I feel that my job gives me new energy* and *I feel that the energy I get from my job exceeds the energy I lose*), a group of individuals (*n* = 220) with an energy-building experience was defined (Table 2).

**Table 2.** Definition of the group with energy-building experience. Number (%) for every combination of answers to two questions

		I feel that my job gives me new energy^a^					
		6	5	4	3	2	1
I feel that the energy I get from my job exceeds the energy I lose^a^	6	**9**	**3**	**1**	0	0	0
		**(1.8)**	**(0.6)**	**(0.2)**	(0)	(0)	(0)
	5	**21**	**35**	**4**	1	0	0
		**(4.3)**	**(7.1)**	**(0.8)**	(0.2)	(0)	(0)
	4	**11**	**83**	**53**	4	0	0
		**(2.2)**	**(16.8)**	**(10.7)**	(0.8)	(0)	(0)
	3	2	34	58	37	2	0
		(0.4)	(6.9)	(11.7)	(7.5)	(0.4)	(0)
	2	0	11	31	29	24	3
		(0)	(2.2)	(6.3)	(5.9)	(4.9)	(0.6)
	1	1	4	9	8	4	12
		(0.2)	(0.8)	(1.8)	(1.6)	(0.8)	(2.4)

a From “Totally agree” (6) to “Totally disagree” (1).

Both single variable analysis and a logistic multivariate regression model were carried out, with energy-building experience as the dependent variable. Explanatory variables included in the model were indices with a bivariate relation (*P* < 0.10) to the dependent variable and a correlation to the other explanatory variables being not too high (*r* < 0.85) according to a collinearity diagnostics (Pallant, 2016). All independent variables were dichotomized as closely as possible to the median value in order to have a neutral split, free from subjective influences. In keeping with the salutogenic perspective of the study, the outcome of the analysis was expressed as positive odds ratio (POR) and 95% confidence interval (CI). The odds ratio was calculated in an ordinary way, but by changing positive and negative outcome in the dependent variable as well as in the explanatory variables (Ejlertsson *et al.*, 2002).

The associations in the regression model were adjusted for age, sex, and employment rate. The significance level was set at 0.05. Statistical analyses were carried out by using SPSS version 22.0.

## Results

The response rate was 84% (*n* = 501). Females were in the majority, 429 (86%) in comparison to 68 men. The sex distribution differed mainly according to profession, 52% of the women were nurses while 75% of the men were physicians. The largest age group was 35–54 years old, and it was dominated by female employees. Most of the employees, 58%, worked full-time (Table 3).

**Table 3.** Description of the respondents, number (%), with regard to age, profession, and working hours in relation to sex.

	Women *n* (%)	Men *n* (%)	Total *n* (%)
**Age**			
34 years and younger	44 (10)	10 (15)	54 (11)
35–54 years	251 (59)	24 (35)	275 (55)
55 years and older	134 (31)	34 (50)	168 (34)
**Profession**			
Physician	48 (11)	51 (75)	99 (20)
Nurse	225 (52)	5 (7)	230 (46)
Administrative staff	81 (19)	1 (1)	82 (16)
Paramedical staff	75 (17)	11 (16)	86 (17)
**Employment rate**			
1–50%	34 (8)	9 (13)	43 (9)
51–80%	146 (34)	18 (26)	164 (33)
81–100%	246 (58)	41 (60)	287 (58)

In total, 91.5% had a positive agreement (4–6 on a 6-step Likert-type scale) with the statement that the energy received or lost at work affects one’s health; of these 23.8% totally agreed (6 on the scale). Correlation coefficients (Pearson) between studied indices were in the range 0.18–0.81 with the highest correlation between work situation and supportive working situation. Self-rated health (SHIS) and energy (index on energy at work) were positively related (*r* = 0.54). Health also correlated highly with internal work experience (*r* = 0.50) and self-esteem/optimism (*r* = 0.59). Report of energy at work was positively correlated (*r* = 0.52–0.64) with supportive working conditions, internal work experiences, recovery, feedback, work situation, and relationship with co-workers.

When energy-building experience was defined as simultaneously agreeing with two statements, if the job gives energy and if that energy exceeds the lost energy (Table 2), the number of employees with an energy-building experience was 220/494 or 44.5%. Spearman correlation coefficients between energy-building experience and the indices ranged from 0.22 to 0.43, with the highest correlation to recovery.

As can be seen from the logistic regression model (Table 4), having an energy-building experience was significantly associated with recovery (POR = 2.78), autonomy (POR = 2.26), positive attributes that characterize the workplace (POR = 2.09), and positive internal work experiences (POR = 1.88). The same four variables were significantly associated with the dependent variable when a regression model using continuous independent variables was built (data not shown).

**Table 4.** Positive odds ratios (POR) with 95% confidence intervals (CI) for predictors of energy-building experience. Results from single variable and multivariable logistic regression

Index^a^	Single variable analyses *n* = 475–489		Multivariable analyses^b^ *n* = 427	
	POR	95% CI	POR	95% CI
Index on recovery	5.29	3.58–7.82	**2.78**	**1.66–4.67**
Autonomy, sub-index on WEMS	3.90	2.67–5.71	**2.26**	**1.39–3.67**
Index on attributes that characterize the workplace	3.91	2.67–5.74	**2.09**	**1.17–3.73**
Internal work experience, sub-index on WEMS	4.08	2.78–6.00	**1.88**	**1.10–3.22**
SHIS	3.17	2.18–4.61	1.58	0.92–2.70
Supportive working conditions, sub-index on WEMS	3.80	2.60–5.57	1.53	0.79–2.96
Index on work situation	2.38	1.64–3.44	1.29	0.69–2.42
Index on expectations	1.95	1.34–2.85	1.13	0.64–1.97
Index on feedback	2.69	1.86–3.88	1.06	0.57–1.97
Index on reflection	2.45	1.70–3.54	1.07	0.64–1.77
Time experience, sub-index on WEMS	2.59	1.79–3.76	1.00	0.60–1.68
Leadership, sub-index on WEMS	2.51	1.73–3.64	0.87	0.51–1.50
Index on relationship with co-workers	2.06	1.39–3.04	0.67	0.38–1.20
Index on self-esteem and optimism	1.81	1.26–2.60	0.67	0.38–1.16

a All indices were used dichotomized according to the median. POR express the odds of the two highest quartiles.

b Hosmer and Lemeshow test *P* = 0.582; Nagelkerke R Square 0.358.

## Discussion

This study is an attempt to further develop the concept of health-promoting workplace. By connecting the theories on regenerative work (Kira, 2003; Ericsson, 2010) and the theories on health promotion (World Health Organization, 1986) a new concept, energy-building experience, is suggested. As was shown in the current study, if a workplace is positive to the extent that the employees experience that the energy they receive is greater than the energy they spend, and if that energy can be associated with employees’ perceived health, we can talk about the concept of energy-building experience at work as an evolution of the current health-promoting workplace.

In our study, 44.5% of the respondents simultaneously agreed with the two statements concerning whether the job gives energy and whether that energy exceeds the lost energy, which was defined as having an energy-building experience. The data showed the tendency that 46% of the women and 38% of the men reported this experience. An energy-building experience was shown to have several main traits. The employees experience (a) possibilities of recovery, (b) high autonomy, (c) a good work situation in terms of feeling comfortable in one’s professional role, meeting with mutual respect, and having a common value system, and (d) they scored high on internal work experiences in terms of feeling that the work is meaningful, feeling happy when going to work and having a diverse and challenging job.

Recovery, including recovery during working hours and outside of work, had the highest relation to having an energy-building experience (POR = 2.78). To reduce the risk of illness and stress, recovery in the form of sleep and rest has been shown to be of great importance (Axelsson *et al.*, 2006; Ekstedt *et al.*, 2009). A study on nurses showed that sleep is an important factor when recovering from work (Silva-Costa *et al.*, 2012). However, there is growing evidence that recovery during working hours is also essential for employees’ perceived health (Hunter and Wu, 2016; Ejlertsson *et al.*, 2018; Sianoja *et al.*, 2018), even though studies on the subject area are limited. One study found that respite interventions can be used to restore energy resources at work (Steidle *et al.*, 2017). Also, it has been shown that the more energy the employees have left at the end of the workday, the better the process of recovery will continue after work (De Bloom *et al.*, 2015).

Experiencing high autonomy had a strong relation to having a energy-building attitude in our study (POR = 2.26). Lack of autonomy can decrease job retention and is an important factor associated with nurse practitioners’ job satisfaction (Han *et al.*, 2015). Previous studies have shown that if the employees are satisfied with autonomy and challenge, they are more likely to report job satisfaction (Katerndahl *et al.*, 2009; Pron, 2013). Sense of usefulness, mastery of work, and zest for work have also been found to be central experiences for remaining at work (Vinje and Ausland, 2013).

A workplace with the characteristics of being positive, safe, and open (POR = 2.09), as well as internal work experiences (POR = 1.88) like coming to work with joy and to experience work as meaningful and challenging, were connected to having an energy-building experience. Work should be enjoyable as well as satisfying and stimulating (World Health Organization, 1986), and joy at work has been confirmed to be a central part of experiencing a good quality of life (Bringsén *et al.*, 2012). A study on nurses showed that spreading a culture of humour in the workplace can improve workplace happiness, which enhances mental, emotional, and physical health (Ghaffari *et al.*, 2015). Moreover, five main themes have been discovered when trying to identify components of a good day at work for nurses. These were: to do something well, to have a good relationship with patients, to feel that you have achieved something, to get the work done, and the fact that you need teamwork (Jackson, 2005), which are factors similar to those presented in the current study.

To the statement that the energy received or lost at work affects one’s health, 91.5% of the respondents had a positive agreement in the current study. However, self-rated health in terms of SHIS did not prove to be an explanatory factor for having an energy-building experience. So, feeling re-energized is important for your health but the relationship does not necessarily apply the other way around. Earlier studies have shown that a necessity for the employees to gain the utmost energy is for the workplace to be healthy (Kroth *et al.*, 2007). Also, to feel recovered and full of energy have been found to be essential factors for sustained working ability in a previous study (Lindberg *et al.*, 2006). One of the most important factors which affect the well-being of the employees is the degree to which they are able to recover from stress and exhaustion at work (Jansen *et al.*, 2002). Studies on employees’ work experiences and health from a salutogenic perspective have shown strong links between working conditions and perceived health (Andersson *et al.*, 2012).

## Strengths and weaknesses

The participation rate was high in the present study, with a representative sample of primary care staff in Sweden. This in combination is important for drawing valid conclusions, as well as reducing selection bias. Also, the possible dropout effects on the findings are small, considering the response rate of 84%. The questionnaire was partly distributed by the managers of the PHCCs which may have had an effect on the response rate as well as influenced the questionnaire answers. Another strength is the high validity. To ensure high content and face validity, the questions used in the questionnaire were developed through individual and focus group interviews, via an expert panel and a pilot study. These questions were then added to the already existing validated instruments SHIS (Bringsén *et al.*, 2009) and WEMS (Nilsson *et al.*, 2010). In terms of internal consistency, all indices used can be considered to have high reliability.

The cross-sectional design of the study prohibits confirming any causality. On the other hand, being the first study exploring energy-building experiences at work, it is possible to draw important conclusions regarding the relationship between experienced health and energy at work. A limitation, in terms of generalizing the results, is the specific work force in primary health care. However, there are many similarities with other work contexts, especially in the health and human service sectors. These are often female dominated and include frequent human contacts and high moral demands. Therefore, we believe that there is a possibility for result transferability to those sectors.

When studying employees’ own experiences, and in this case energy-building experiences, there is always a risk of not considering all influencing factors, such as lifestyle, household arrangement, and ongoing morbidity. However, cofounders like sex, age, and working time were adjusted for. The risk of the healthy worker effect should also be mentioned as a limitation when performing research in the work context, as an individual must be relatively healthy to be employable. Since the results were obtained from self-report, it may include some information bias. The questionnaire itself, however, with its short recall time, may reduce this bias. Also, we recognize that there is no distinct definition of concepts like energy and recovery. Therefore, the respondents’ interpretations of the concepts can differ between individuals and their different experience of work demands. Finally, the social desirability of participating might also introduce some bias.

## Concluding remarks

In conclusion, the results of the present study showed that it is possible for employees to have an energy-building experience at work. This is valuable information regarding future workplace health-promotion efforts and for further development of the concept of regenerative work. For an increased energy experience at work, and thereby better health among the employees, there are four factors that need to be focused on. First and foremost, recovery, but also autonomy, workplace characteristics, and positive internal work experience. By using existing research, some of these energy promoting factors can be put into practice right away. However, additional research of the areas is important to develop work practice and organization further. Recovery at work is currently being further illuminated in an intervention study, where different recovery activities are integrated into daily work at several workplaces to explore if and how this will influence the employees’ experience of recovery. If this intervention succeeds, the knowledge may contribute to creating energy-building workplaces in similar work contexts.
